# Validity and reliability of an accelerometer-based player tracking device

**DOI:** 10.1371/journal.pone.0191823

**Published:** 2018-02-08

**Authors:** Daniel P. Nicolella, Lorena Torres-Ronda, Kase J. Saylor, Xavi Schelling

**Affiliations:** 1 Southwest Research Institute, San Antonio, Texas, United States of America; 2 Institute of Sport, Exercise and Active Living, College of Sport and Exercise Science, Victoria University, Melbourne, VIC, Australia; Universita degli Studi di Verona, ITALY

## Abstract

This study aimed to determine the intra- and inter-device accuracy and reliability of wearable athletic tracking devices, under controlled laboratory conditions. A total of nineteen portable accelerometers (Catapult OptimEye S5) were mounted to an aluminum bracket, bolted directly to an Unholtz Dickie 20K electrodynamic shaker table, and subjected to a series of oscillations in each of three orthogonal directions (front-back, side to side, and up-down), at four levels of peak acceleration (0.1g, 0.5g, 1.0g, and 3.0g), each repeated five times resulting in a total of 60 tests per unit, for a total of 1140 records. Data from each accelerometer was recorded at a sampling frequency of 100Hz. Peak accelerations recorded by the devices, Catapult PlayerLoad™, and calculated player load (using Catapult’s Cartesian formula) were used for the analysis. The devices demonstrated excellent intradevice reliability and mixed interdevice reliability. Differences were found between devices for mean peak accelerations and PlayerLoad™ for each direction and level of acceleration. Interdevice effect sizes ranged from a mean of 0.54 (95% CI: 0.34–0.74) (small) to 1.20 (95% CI: 1.08–1.30) (large) and ICCs ranged from 0.77 (95% CI: 0.62–0.89) (very large) to 1.0 (95% CI: 0.99–1.0) (nearly perfect) depending upon the magnitude and direction of the applied motion. When compared to the player load determined using the Cartesian formula, the Catapult reported PlayerLoad™ was consistently lower by approximately 15%. These results emphasize the need for industry wide standards in reporting validity, reliability and the magnitude of measurement errors. It is recommended that device reliability and accuracy are periodically quantified.

## Introduction

Wearable devices designed to measure performance metrics have become ubiquitous in the athletics community. These devices are targeted to sports scientists, trainers, coaches, and athletes, as a means by which training regimens can be adjusted to maximize performance gains and reduce the risk of injury (for review see [1, 2]).

Over more than a decade, Global Positioning System (GPS)-based wearable-tracking devices have been widely used to monitor outdoor training allowing a better understanding of the physical requirements of sport while being less time-consuming than traditional time-motion analysis [3]. Furthermore, these devices provide for real-time movement analysis and feedback that can be directly incorporated into the training regime. Nevertheless, the use of GPS-based devices presents certain limitations: a) they cannot be used indoors [3], and b) questionable validity and reliability to accurately assess short, high-intensity movements due to its low sampling rate (1–10 Hz) [3]. Another common approach to player tracking is the use of multi-camera systems that use image-processing techniques (i.e., motion capture) to determine the position of an athlete within a particular physical space. This approach is desirable because it is less invasive to the athlete as it does not require the use of a wearable device. Advances in computer processing (e.g., software algorithms and hardware) continue to make this approach more and more desirable, but the currently available systems suffer from two limitations: a) their use is generally confined to indoor sports because tracking accuracy depends upon the size of the physical space, and b) these systems require post-event processing to accurately determine player position.

Another approach that has gained acceptance within the area of player tracking is wearable, acceleration-based tracking devices that incorporate microelectromechanical systems (MEMS) gyroscopes, magnetometers, and accelerometers into a single player-worn unit [4]. The devices utilize tri-axial accelerometers that are not positional based, but movement based (anterior-posterior, medial-lateral, and vertical) [5], to obtain descriptors of sports activities, such us accelerations, decelerations, jumps, change of direction or other accelerometer-derived measurements [6]. One such derived measurement is the PlayerLoad™ (Catapult Innovations, Melbourne, Australia), which is used to describe and quantify an athlete’s external workload [7–10].

Within the sports community, there lacks a governing body responsible for defining and maintaining standards for such devices. Therefore, it is the responsibility of the practitioners (e.g., sports scientists) to determine both the validity and accuracy of data provided by these devices. Furthermore, without defined procedures and reports by the manufacturers that can be used by the final consumer to replicate and quantify the accuracy of wearable devices, the users must either take accuracy claims at face value, or must conduct their own experiments to verify these claims.

Unquestionably, technology presents a great opportunity to obtain real time data. However, it is imperative to understand the specificity, validity and reliability of the devices that one desires to implement in managing athlete training and competition environments.

Thus, the aim of this study was to determine the accuracy and reliability of wearable athlete tracking devices under controlled laboratory conditions subjected to a series of highly controlled laboratory-based sinusoidal motions implemented through a shaker table used in environmental testing of electronic instruments.

## Materials and methods

**Fig 1:**
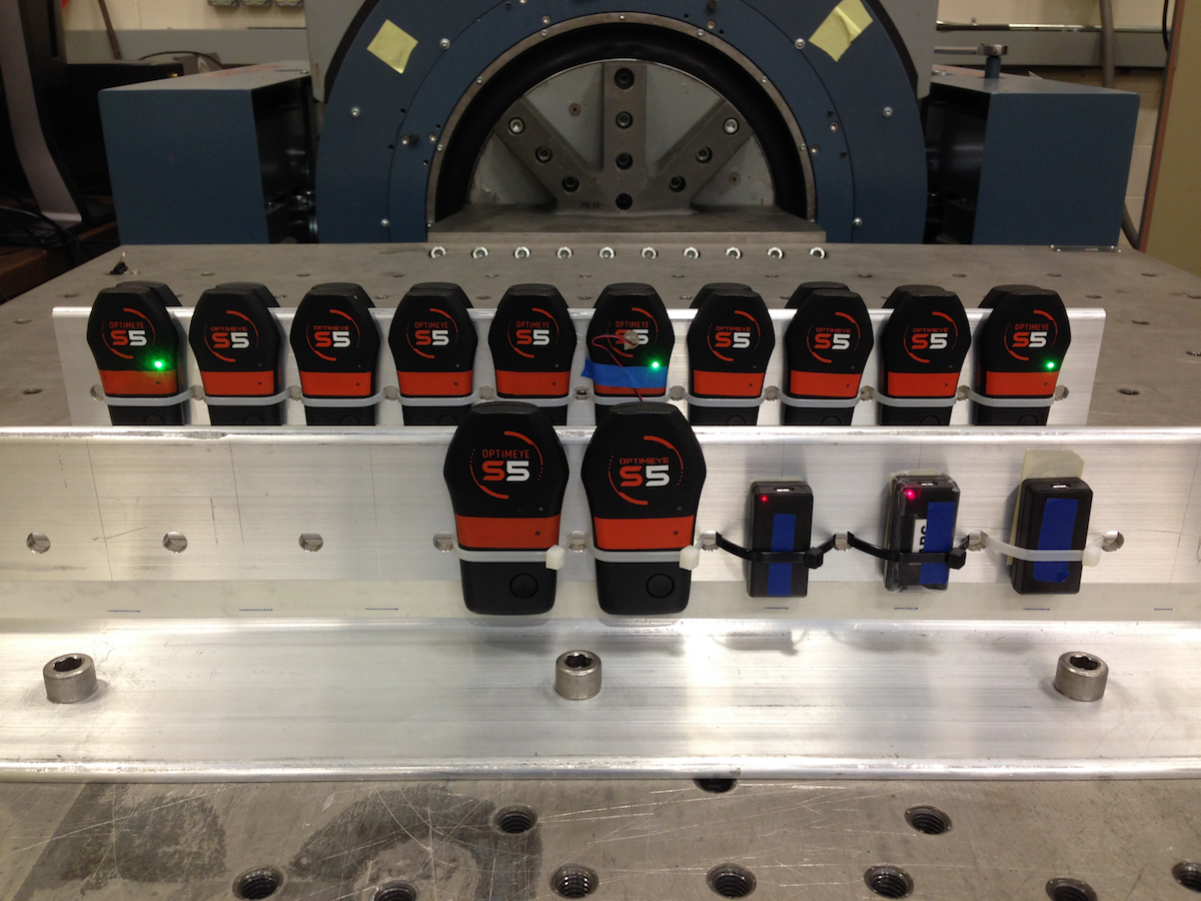
Accelerometer mounting bracket and shaker table configuration. The shaker table motion is in the fore-aft direction in this photo. As attached, the devices are subjected to motion in the X direction (front-back). The mounting bracket is rotated 90 degrees on the shaker table for Y direction motion (side-side). For Z direction motion, the table is removed, the drive motor is rotated 90 degrees, and the mounting bracket is attached directly to the drive motor output mount.

A total of nineteen portable accelerometers (Catapult OptimEye S5, Catapult Innovations, Team Sport 5.0, Melbourne, Australia) were analyzed in this study. Each accelerometer was identically and rigidly mounted to aluminum brackets using double sided tape and plastic cable ties. The aluminum brackets were bolted directly to an Unholtz Dickie 20K electrodynamic shaker table (Fig 1) capable of generating controlled oscillations at frequencies up to 20 Hz. This shaker table is used as a gold standard in environmental testing of electronic instruments such as NASA microsatellites. Along with the nineteen portable accelerometers, a calibrated single-axis reference accelerometer (J353B31, PCB Piezoelectronics, Depew, NY) was also mounted to the shaker table to serve as a reference accelerometer. The devices were then subjected to a series of oscillations in each of three orthogonal directions (x-front-back, y-side-to-side, and z-up-down). For each direction, the devices were subjected to a series of highly controlled laboratory-based sinusoidal motions of four combinations of oscillation frequencies resulting in peak accelerations of 0.1g, 0.5g, 1.0g, and 3.0g in each of three orthogonal directions (Table 1). Each test was repeated 5 times, for 30s for each acceleration in each direction (x, y, and z), resulting in a total of 60 tests per unit, and a total of 1140 records.

**Table 1:** Accelerometer test matrix.

Frequency (Hz)	Displacement (m)	Velocity (m/sec)	Acceleration (g)
2.0	0.012	0.078	0.100
3.0	0.027	0.260	0.500
4.0	0.031	0.390	1.000
8.0	0.023	0.585	3.000

Data from each accelerometer was recorded at a sampling frequency of 100Hz and downloaded from each device using either the manufacturer’s software and firmware (Catapult Sprint Version 5.1.7, Melbourne, Australia, firmware Version 7.17). The acceleration data from each unit was saved to a SQLite (public domain SQL database engine) database using custom data extraction software written in Python (v.2.7). The time vs. acceleration data for each device for each individual test was saved to the database. For the Catapult units, the reported instantaneous PlayerLoad™ was also saved. PlayerLoad™ is a measurement provided by Catapult Innovations software, and is an arbitrary unit defined as an “instantaneous rate of change of acceleration divided by a scaling factor.” The instantaneous rate of change of acceleration is commonly known in physics as “jerk” and is defined as:

$$\vec{j}=\frac{d\vec{a}(t)}{dt}=\vec{a}(t)=\frac{d^{2}\vec{v}(t)}{d(t^{2})}=\ddot{\vec{v}}=\frac{d^{3}\vec{\gamma }(t)}{d(t^{3})}=\dddot{\vec{\gamma ,}}$$ (1)

where $\vec{a}$ is acceleration, $\vec{v}$ is velocity, $\vec{\gamma }$ is position, and *t* is time. Utilizing accelerometers within the three planes of movement to quantify movement intensity [4] PlayerLoad™ is calculated as follows:

$$\mathrm{PlayerLoad}™=\sqrt{\frac{{\left(a_{y(t)}-a_{y(t-1)}\right)}^{2}+{\left(a_{x(t)}-a_{x(t-1)}\right)}^{2}+{\left(a_{z(t)}-a_{z(t-1)}\right)}^{2}}{100}},$$ (2)

where a_y_ is forward (anterior-posterior) acceleration, a_x_ is sideways (medial-lateral) acceleration, and a_z_ is vertical acceleration. Once the data from each unit was saved in the database, a series of Python scripts were written to process the data for this study.

Using a peak detection algorithm [9], average peak maximum and minimum accelerations were determined over a 10s time interval from the middle of each 30s test run to avoid any possible bias in the data caused by the initial or final motion of shaker table. In addition to peak accelerations, we captured the instantaneous Catapult PlayerLoad™ reported by the OptimEye S5 units and independently calculated the player load by applying the same Cartesian formula (Eq 2) to the accelerations reported by the units. Likewise, player load was calculated for the the PCB reference accelerometer using the same formula.

### Statistical analysis

Descriptive statistics are expressed as means and standard deviations (SD) and coefficient of variation (CV) of the five trials for each accelerometer for each direction and acceleration magnitude. The CV was rated as good when CV <5%, as moderate when CV was 5–10% and as poor when CV was >10% [11]. The uncertainty was expressed as 95% confidence interval (CI). Within device (intra-unit), test-retest reliability was evaluated for each loading direction and magnitude using the intraclass correlation coefficient (ICC) where < 0.1 trivial, 0.10–0.29 small, 0.30–0.49 moderate, 0.50–0.69 large, 0.70–0.89 very large, and >0.90 nearly perfect [12]. Between device (inter-unit) reliability for mean measured peak accelerations and the Catapult determined PlayerLoad™ data was analyzed using a one-way ANOVA with device as the factor for each direction and magnitude of oscillatory loading. A post-hoc pair-wise analysis was performed using Tukey’s honestly significant difference (HSD). Between device effect size was determined as Cohen’s d where 0–0.19 was considered trivial, 0.2–0.59 small, 0.6–1.19 moderate, 1.20–1.99 large, 2.00–3.99 very large, and d≥4 extreme [12]. Statistical analyses were performed using JMP (JMP v. 12, SAS Institute, Cary, NC).

## Results

The results for the mean peak accelerations and Catapult derived instantaneous PlayerLoad™ values for each direction and level of acceleration CV ranged from 0.01% to <3.0%, with the majority of CV values <1.0% (see Table 2 for X axis 3g, 8 Hz conditions), indicating excellent intradevice reliability [4,10]. ICCs ranged from 0.77 (95% CI: 0.62–0.89) (very large) to 1.0 (95% CI: 0.99–1.0) (nearly perfect) (Table 3).

**Table 2:** Descriptive statistics: 3.0 g, 8 Hz applied peak acceleration. Data for 0.1g, 0.5g and 1.0g applied acceleration can be found in the Supporting Information.

3.0 g	X (forward-back)						Y (side-side)						Z (up-down)					
	Peak		Catapult PL		Calculated PL		Peak		Catapult PL		Calculated PL		Peak		Catapult PL		Calculated PL	
Accelerometer	Mean±SD	CV (%)	Mean±SD	CV (%)	Mean±SD	CV (%)	Mean±SD	CV (%)	Mean±SD	CV (%)	Mean±SD	CV (%)	Mean±SD	CV (%)	Mean±SD	CV (%)	Mean±SD	CV (%)
C1	2.99±0.07	0.07	818.17±0.08	0.08	972.45±0.08	0.08	3.06±0.09	0.09	810.68±0.02	0.02	942.28±0.40	0.40	4.04±0.09	0.09	812.33±0.06	0.06	949.58±0.54	0.54
C2	3.00±0.05	0.05	811.73±0.02	0.02	965.63±0.05	0.05	3.06±0.05	0.05	808.93±0.02	0.02	949.82±0.49	0.49	3.99±0.05	0.05	799.91±0.02	0.02	937.26±0.38	0.38
C3	2.90±2.57	2.57	782.15±2.58	2.58	930.13±2.50	2.50	2.94±2.85	2.85	776.33±2.99	2.99	903.70±2.94	2.94	3.98±0.03	0.03	798.26±0.04	0.04	934.24±0.50	0.50
C4	2.99±0.02	0.02	815.57±0.04	0.04	969.72±0.06	0.06	3.07±0.05	0.05	813.91±0.05	0.05	953.64±0.35	0.35	3.99±0.03	0.03	800.27±0.06	0.06	918.50±0.73	0.73
C5	3.02±0.06	0.06	814.02±0.03	0.03	967.97±0.09	0.09	2.99±0.04	0.04	813.14±0.02	0.02	966.56±0.06	0.06	3.99±0.06	0.06	799.54±0.03	0.03	946.55±0.16	0.16
C6	3.12±0.25	0.25	824.65±0.04	0.04	980.19±0.05	0.05	3.00±0.30	0.30	815.90±0.05	0.05	954.16±0.49	0.49	4.01±0.10	0.10	804.76±0.05	0.05	952.84±0.25	0.25
C7	3.06±0.06	0.06	810.85±0.04	0.04	964.32±0.04	0.04	2.99±0.04	0.04	811.27±0.02	0.02	941.80±0.63	0.63	4.01±0.05	0.05	804.63±0.04	0.04	949.66±0.43	0.43
C8	2.99±0.10	0.10	807.14±0.03	0.03	959.17±0.06	0.06	3.10±0.08	0.08	814.77±0.06	0.06	944.04±0.27	0.27	3.99±0.18	0.18	802.84±0.06	0.06	946.41±0.10	0.10
C9	2.98±1.76	1.76	796.67±1.76	1.76	947.42±1.71	1.71	2.94±2.20	2.20	798.56±2.04	2.04	945.72±1.97	1.97	3.99±0.09	0.09	797.41±0.09	0.09	939.89±0.63	0.63
C10	2.76±4.65	4.65	756.36±4.77	4.77	900.59±4.73	4.73	2.92±5.00	5.00	759.94±5.20	5.20	888.67±5.38	5.38	4.01±0.11	0.11	801.58±0.08	0.08	936.66±0.75	0.75
C11	3.01±0.23	0.23	813.21±0.08	0.08	967.53±0.09	0.09	2.96±0.27	0.27	812.63±0.06	0.06	966.37±0.06	0.06	3.96±0.22	0.22	796.50±0.09	0.09	937.26±0.22	0.22
C12	3.00±0.14	0.14	809.74±0.07	0.07	963.13±0.11	0.11	3.13±0.18	0.18	815.24±0.08	0.08	969.51±0.08	0.08	4.00±0.20	0.20	801.54±0.03	0.03	937.51±0.38	0.38
C13	2.88±2.61	2.61	783.71±2.82	2.82	931.99±2.79	2.79	2.97±3.01	3.01	784.14±3.18	3.18	855.74±3.34	3.34	4.01±0.24	0.24	800.60±0.08	0.08	930.94±0.58	0.58
C14	2.99±0.08	0.08	815.19±0.02	0.02	969.60±0.07	0.07	3.09±0.02	0.02	815.27±0.07	0.07	951.85±0.42	0.42	4.01±0.10	0.10	800.40±0.04	0.04	933.23±0.28	0.28
C15	3.03±0.27	0.27	814.24±0.05	0.05	966.79±0.14	0.14	2.99±0.23	0.23	814.11±0.03	0.03	967.65±0.11	0.11	3.99±0.23	0.23	801.55±0.06	0.06	946.55±0.28	0.28
C16	2.95±0.10	0.10	809.76±0.03	0.03	962.55±0.08	0.08	3.04±0.03	0.03	808.05±0.05	0.05	961.05±0.04	0.04	3.99±0.03	0.03	801.68±0.05	0.05	933.93±0.43	0.43
C17	6.91±0.24	0.24	1596.92±0.09	0.09	1897.58±0.09	0.09	2.99±0.27	0.27	811.56±0.06	0.06	965.36±0.13	0.13	4.00±0.24	0.24	806.25±0.08	0.08	959.99±0.15	0.15
C18	2.93±0.24	0.24	802.02±0.05	0.05	952.41±0.07	0.07	3.05±0.13	0.13	808.87±0.06	0.06	945.96±0.36	0.36	3.95±0.21	0.21	794.99±0.06	0.06	918.81±0.53	0.53
C19	3.00±0.24	0.24	809.23±0.10	0.10	962.36±0.04	0.04	2.96±0.22	0.22	811.07±0.05	0.05	963.96±0.12	0.12	3.97±0.18	0.18	799.12±0.10	0.10	942.72±0.30	0.30
ref	3.03±0.04	0.04			955.64±0.10	0.10	3.01±0.25	0.25			952.96±0.14	0.14	2.97±0.62	0.62			939.17±0.62	0.62

PL = Player Load, C1-C19, Catapult OptimEye S5, Ref = PCB reference accelerometer

**Table 3:** Intraclass correlation coefficients (ICC).

Direction	x				y				z			
acceleration	0.1	0.5	1.0	3.0	0.1	0.5	1.0	3.0	0.1	0.5	1.0	3.0
ICC	1.0	1.00	1.00	1.00	1.0	1.0	0.96	0.77	1.0	1.0	1.0	1.0
95% CI	0.99–1.0	0.99–1.0	0.99–1.0	0.99–1.0	0.99–1.0	0.99–1.0	0.92–0.98	0.62–0.89	1.0–1.0	1.0–1.0	1.0–1.0	1.0–1.0

**Fig 2:**
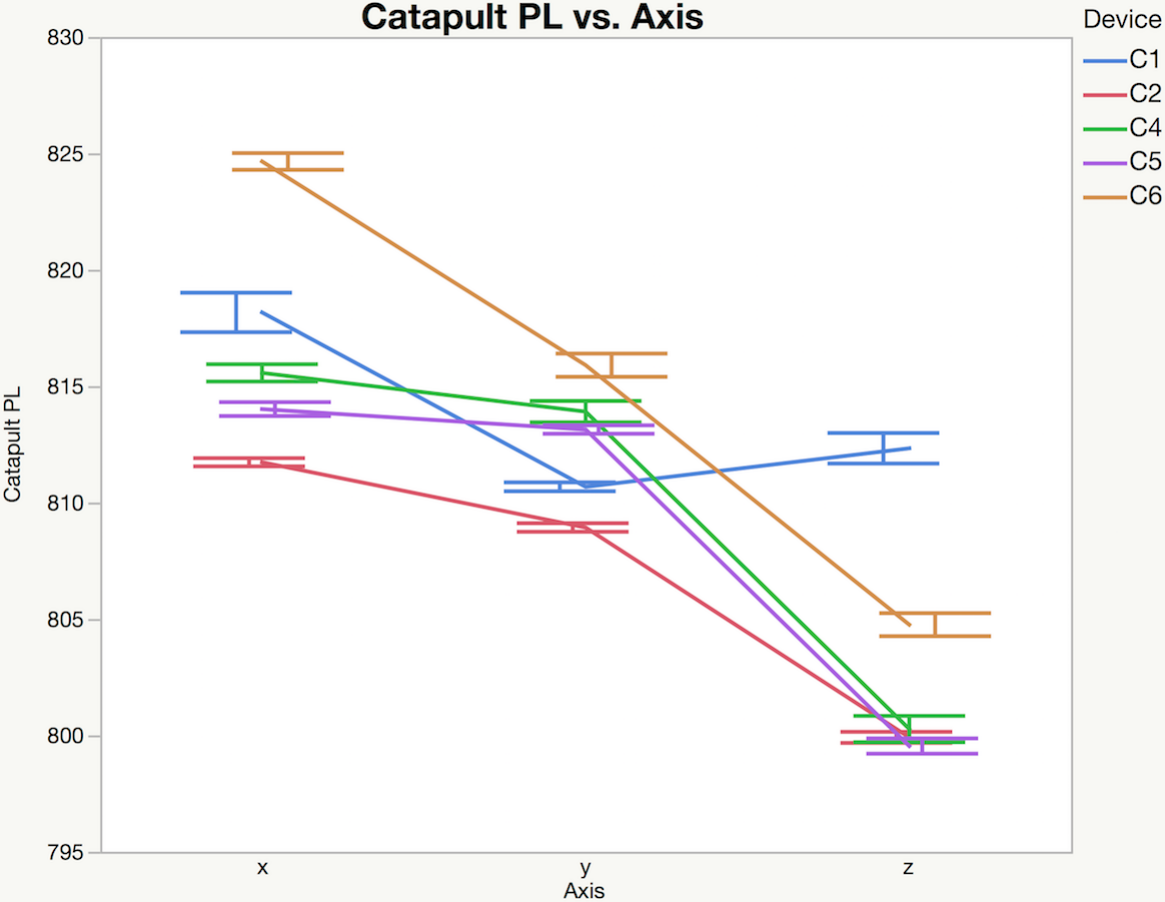
Mean catapult PlayerLoad™ (PL) from five repeats with 95% confidence intervals. For clarity only 5 devices are shown (3g peak acceleration, 8 Hz). Please see Supporting Information for complete data set (S1 Data).

**Fig 3:**
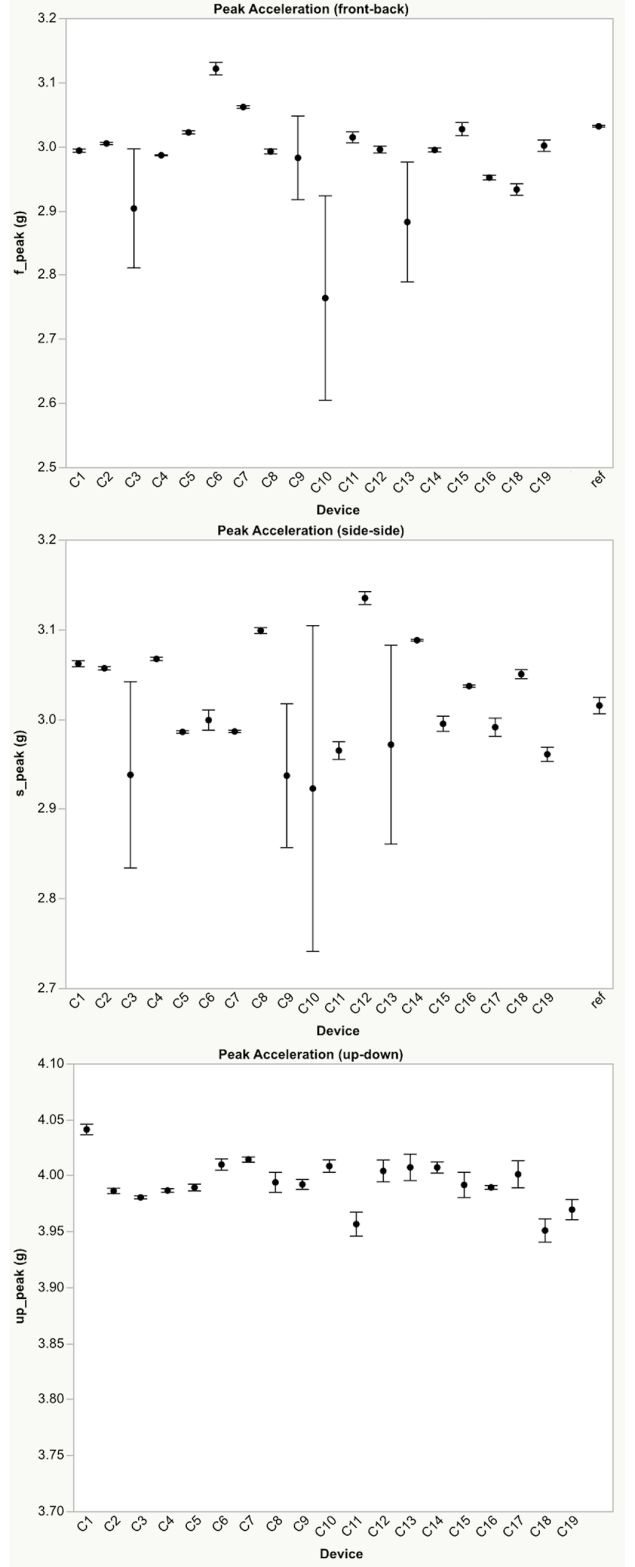
Peak accelerations. Mean ±95% confidence intervals peak acceleration measured by each device resulting from oscillatory motion applied in the top) x-direction (front-back), middle) y-direction (side-side), and bottom) z-direction (up-down) with 3g peak acceleration (8 Hz). Note: Data from device C17 was an outlier and was removed from the x-direction plot. The reference accelerometer is gravity corrected and is not shown on the z-direction plot. Please see Supporting Information for complete data set (S1 Data).

**Fig 4:**
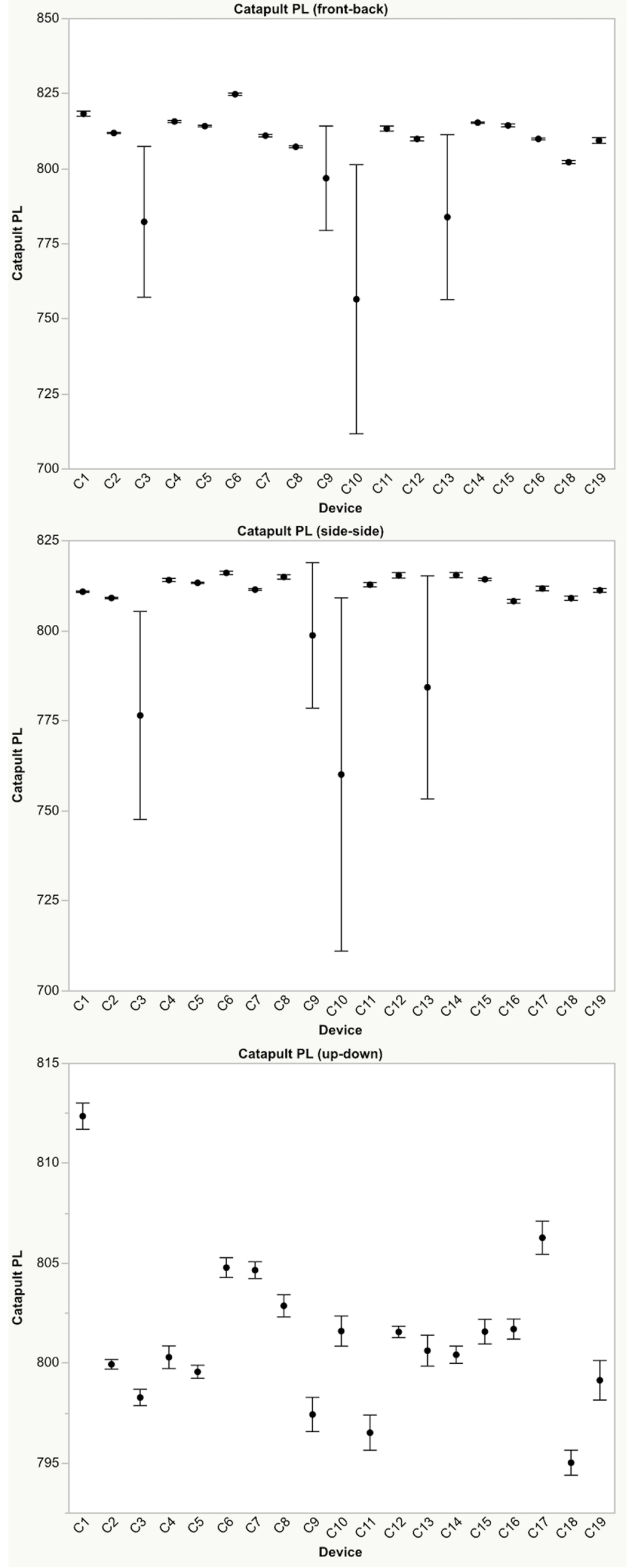
Catapult PlayerLoad™. Mean ±95% confidence intervals reported by each device resulting from oscillatory motion applied in the top) x-direction (front-back), middle) y-direction (side-side), and bottom) z-direction (up-down) with 3g peak acceleration (8 Hz). Note: Data from device C17 was an outlier and was removed from the x-direction plot.

Differences were found between devices for mean peak accelerations, and PlayerLoad™ for each direction and level of applied external loading (Figs 2–4). PlayerLoad™ interdevice effect sizes (Cohen’s d) ranged from a mean of 0.54 (95% CI: 0.34–0.74) (small) to 1.20 (95% CI: 1.08–1.30) (large) depending upon the magnitude and direction of the applied motion (Table 4). Differences were also found between Catapult peak accelerations and the PCB reference accelerometer peak accelerations for each direction and level of applied acceleration (Fig 4), where effect sizes ranged from a mean of 0.36 (95% CI: 0.21–0.52) (small) to 1.15 (95% CI: 1.02–1.27) (moderate) (Table 5). The mean percent difference between the reference accelerometer measured peak acceleration and the Catapult device measured peak acceleration ranged from 23.5% to 1.0% (Table 6). The full test dataset is available in the Supporting Information section online.

**Table 4:** Catapult player Load^™^ interdevice effect sizes (Cohen’s d).

direction	x				y				z			
acceleration (g)	**0.1**	**0.5**	**1.0**	**3.0**	**0.1**	**0.5**	**1.0**	**3.0**	**0.1**	**0.5**	**1.0**	**3.0**
Mean	0.54	1.00	1.07	0.99	0.77	1.00	0.90	0.85	1.15	0.99	1.10	1.11
Std Dev	1.33	0.81	0.81	0.84	1.21	1.01	0.95	0.89	0.79	1.05	0.90	0.89
Upper 95% Mean	0.74	1.13	1.20	1.12	0.95	1.16	1.04	0.98	1.27	1.15	1.24	1.25
Lower 95% Mean	0.34	0.87	0.94	0.85	0.59	0.84	0.75	0.71	1.02	0.83	0.97	0.98
CV	245.28	81.33	75.86	85.53	157.66	100.69	106.27	105.71	69.20	106.33	81.48	79.56

**Table 5:** Peak acceleration interdevice effect sizes (Cohen’s d).

direction	x				y				z			
acceleration (g)	**0.1**	**0.5**	**1.0**	**3.0**	**0.1**	**0.5**	**1.0**	**3.0**	**0.1**	**0.5**	**1.0**	**3.0**
Mean	1.20	0.67	1.12	1.08	1.06	1.13	1.14	1.06	0.36	1.17	1.16	0.59
Std Dev	0.81	1.28	0.89	0.82	0.99	0.90	0.84	0.75	1.02	0.84	0.82	1.32
Upper 95% Mean	1.30	0.82	1.25	1.20	1.20	1.26	1.26	1.17	0.52	1.30	1.29	0.79
Lower 95% Mean	1.08	0.52	0.98	0.97	0.91	0.99	1.02	0.95	0.21	1.04	1.04	0.39
CV	67.54	190.72	79.74	76.01	93.43	80.05	73.69	71.08	279.23	71.80	70.68	225.29

**Table 6:** Accuracy of wearable devices compared to the reference accelerometer (percent difference).

	0.1g	0.5g	1.0g	3.0g
**X-direction**	22.3%	4.1%	2.6%	2.4%
**Y-direction**	23.5%	4.8%	2.6%	1.8%
**Z-direction**	4.9%	1.9%	1.0%	1.0%

**Fig 5:**
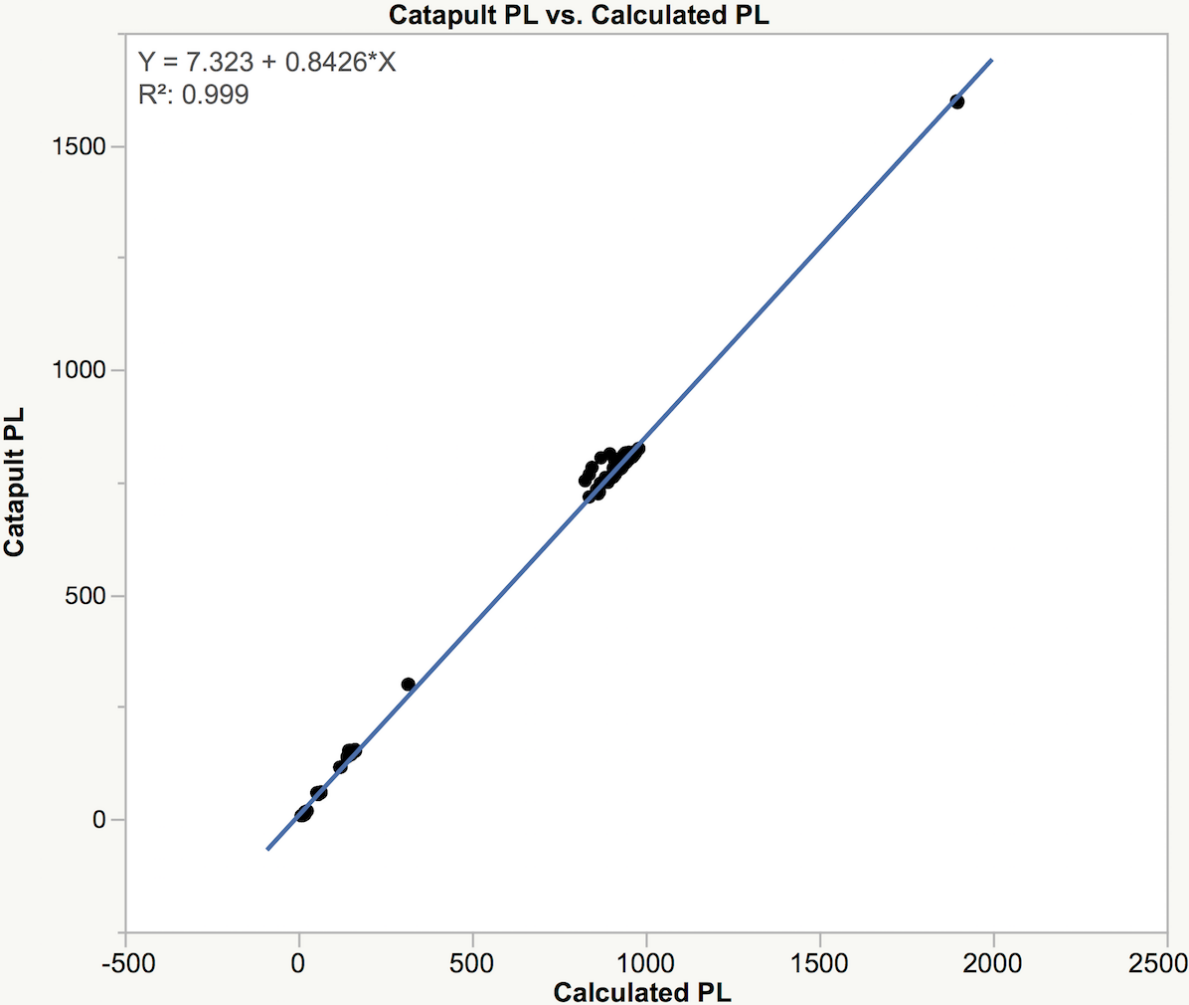
Catapult PlayerLoad™ vs. calculated player load.

When compared to the player load determined using the Cartesian formula (Eq 1), the Catapult reported PlayerLoad™ is consistently lower by approximately 15% (Fig 5).

## Discussion

The objective of this investigation was to quantify both the reliability and validity of a commercially available wearable inertial measuring unit used for athletic tracking and performance analysis. The devices demonstrated excellent intradevice reliability and mixed interdevice reliability depending on the direction and magnitude of the applied accelerations. Similarly, the devices demonstrated mixed accuracy when compared to the reference accelerometer with effects sizes ranging from trivial to small. A secondary objective was to compare PlayerLoad™ vs a calculated player load determined using the Cartesian formula reported by the manufacturer.

Differences were found between devices for both mean PlayerLoad™ and mean peak accelerations with effect sizes ranging from trivial to extreme, depending on individual units (Figs 2–4). To quantify device validity, the peak accelerations measured by each device was compared to peak accelerations measured using a calibrated reference accelerometer attached to the shaker table. Similarly, differences were found between the mean OptimEye S5 measured peak accelerations and the mean peak accelerations measured by the reference accelerometer, with mean effect sizes ranging from trivial at higher loading levels (0.5g - 3.0g) to extreme at the lowest loading level (0.1g) depending upon the unit (Fig 4).

Previous investigations have quantified the reliability and validity of accelerometers for use in tracking subject activity [13–15]. Following a similar approach to the method described herein, Boyd et al. [4], using a hydraulic universal testing machine to oscillate devices at specified acceleration ranges, reported within- and between- device CVs of ≤1.10% for device reported PlayerLoad™ although they did not report device validity. Using a controlled laboratory based impact testing protocol, Kelly et al. [11] reported no significant within device differences (CV’s between 1.87% and 2.21%), but did find significant differences between the device-reported mean peak accelerations and mean peak accelerations reported by a calibrated PCB reference accelerometer (percent differences ranging from 32% to 35%). Similarly, using a shaker table to apply controlled, repeatable motion, Kransoff et al. [12] found excellent within device reliability (CVs less than 0.6%), but poor between device reliability (CVs between 9.5% and 34.7%).

The Catapult devices generally showed excellent intra-device reliability with the majority of within device CVs less than 2.0%. However, differences were found between devices with larger variability measured in the x-, and y-directions compared to the z-direction suggesting a possible device calibration discrepancy (Fig 2). In general, smaller between device reliability effect sizes were seen when motion was applied in the z-direction (device up-down) compared to the x- (device front-back) and y-directions (device side-side) (Figs 3 and 4). Similarly, Barrett and colleagues [16] investigated the reliability of the PlayerLoad™ while running on a treadmill, reporting that the between-device variability in PlayerLoad™ was plane dependent (9.1% in x-directions, 12.0% in y-direction and 6.3% in z-direction). Based on these results, caution should be taken when comparing PlayerLoad™ or mean peak acceleration between devices, especially when partitioning the results by planes of motion.

Since the reliability between devices is plane and acceleration magnitude dependent, the accuracy and reliability of metrics based on acceleration-based thresholds such as the Inertial Movement Analysis (IMA) provided by Catapult [17] may be unreliable, especially when used to assess unpredictable, multiplane, high-intensity actions (accelerations, decelerations, change of direction, rotations, jumps, contacts, etc.). This hypothesis is supported by previous reports on inertial movement analysis while walking, jogging, running, changing direction, or jumping, which found low IMA reliability during high-intensity running and sport-specific movements [18, 19]. Therefore, there is a need for further research to determine appropriate filters, thresholds settings, and algorithms to detect events in order to properly analyze inertial movement.

When comparing the results from the Catapult PlayerLoad™ and calculated player load, we found that PlayerLoad™ is consistently lower by approximately 15%, suggesting that data filtering techniques affect the Catapult reported results. Similar to previously reported GPS player tracking results, due to the inherent noise in the raw data, manufacturers routinely apply different filtering techniques to smooth velocity and acceleration data [20]. This becomes problematic if the practitioner does not know the algorithms used by the manufacturers to process the raw data. The effect of data filtering as well as periodic updates to the device firmware alters the device outputs, potentially compromising results and making it difficult to perform historical comparisons [4]. As recently reported [20], it is unknown how changes to data filtering, ‘dwell time,’ or minimum effort duration will directly affect the reported athlete performance measures. Therefore, the filtering techniques applied to the raw data, the device settings, device firmware, and software version used during the data collection should be reported both by the manufacturer and when studies are reported in the literature allowing for both more equitable comparisons between studies and reproducibility of the research.

The methods used in the present investigation can be utilized to provide a baseline assessment of reliability and validity of wearable devices whose intended use is to quantify measures of athlete physical performance. This method employs the application of highly-controlled, laboratory-based, applied oscillatory motion, and will provide a repeatable, verified, applied motion validated using a calibrated reference accelerometer. This type of controlled laboratory testing can allow for the determination of the limits of performance, reliability, and validity of devices employed to evaluate physical performance. While this characterization method provides a performance baseline, the use of these devices in an applied setting typically involves placing the device in a vest worn by the athlete. As such, the interaction and relative movement of each device with the vest and the interaction and relative movement of the vest with the athlete will introduce an additional level of variability in the device recorded data. Further investigation is required to accurately characterize these interactions in order to provide a more complete description of overall device application variability.

As the use of wearable devices becomes more ubiquitous, standard methods of device reported data verification and validation should be required. Standard test methods with calibrated reference devices should be used as a basis of comparison to device reported measures. Also, since one of the units had to be removed from the study as it was an outlier, and several devices showed poor between-device reliability, we recommend periodic device calibration in order to minimize the error of measurement and to identify malfunctioning units. A possible limitation of the present study is that while the experimental protocol was designed to minimize extraneous vibrations and off-axis error, sources of error could include variations in device hardware including accelerometer sensitivities and orientation of sensors within the device. In addition, slight misalignments of the attachment of the devices to the shaker table could result in small variations in reported accelerations and derived PlayerLoad™ metrics.

Future research should focus on controlled multi-plane movements, with prescribed kinematics using methods that allow for the replication of typical sports movements such us linear sprints, change of directions, jumps, as well as sport specific movements.

## Conclusions

The Catapult OptimEye S5 units showed excellent intradevice reliability. The data collected with these devices, and therefore possible decisions made using this data, will be reliable when the same device is used for each athlete over the time course of interest. However, since the interdevice reliability was shown to be highly variable (trivial to extreme), data collected on individual athletes using different devices will be of diminished reliability and utility. Therefore, it is recommended that the same device be used for the same athlete over the course of a season (or longer) in order to provide a consistent basis for comparison and to avoid interdevice variability affecting the collected data. Furthermore, acceleration data from the OptimEye S5 devices differed from data reported by the calibrated reference accelerometer at the lower acceleration and frequency test conditions (0.1g, 2 Hz). Thus, if the activity of interest includes a significant amount of movement within this range, the captured data will be less reliable and therefore less useful. Finally, player load data computed from the OptimEye S5 using the Catapult published PlayerLoad™ formula (Eq 2) is significantly biased when compared to the Catapult reported PlayerLoad™, indicating additional data manipulation occurs prior to data output that is not described by the manufacturer.

Given the results of the present study it is recommended that wearable devices undergo periodic reliability and validity assessments using calibrated and well defined methods. As such, we recommend that the community develop and adopt device verification, validation, testing, and reporting standards so that practitioners can reliably, confidently, and interchangeably use devices from various manufactures in their programs.

## Supporting information

S1 Data Shaker table tests data file. (XLSX)
